# Effect of *Lactobacillus plantarum* Strain K21 on High-Fat Diet-Fed Obese Mice

**DOI:** 10.1155/2015/391767

**Published:** 2015-02-23

**Authors:** Chien-Chen Wu, Wei-Lien Weng, Wen-Lin Lai, Hui-Ping Tsai, Wei-Hsien Liu, Meng-Hwan Lee, Ying-Chieh Tsai

**Affiliations:** ^1^Institute of Biochemistry and Molecular Biology, National Yang-Ming University, Taipei 11221, Taiwan; ^2^Probiotics Research Center, National Yang-Ming University, Taipei 11221, Taiwan; ^3^School of Medical Laboratory and Biotechnology, Chung Shan Medical University, Taichung 40201, Taiwan; ^4^Clinical Laboratory, Chung Shan Medical University Hospital, Taichung 40201, Taiwan; ^5^Division of Animal Technology, Animal Technology Laboratories, Agricultural Technology Research Institute, Zhunan Township, Miaoli County 35053, Taiwan

## Abstract

Recent studies have demonstrated beneficial effects of specific probiotics on alleviating obesity-related disorders. Here we aimed to identify probiotics with potential antiobesity activity among 88 lactic acid bacterial strains via *in vitro* screening assays, and a *Lactobacillus plantarum* strain K21 was found to harbor abilities required for hydrolyzing bile salt, reducing cholesterol, and inhibiting the accumulation of lipid in 3T3-L1 preadipocytes. Furthermore, effects of K21 on diet-induced obese (DIO) mice were examined. Male C57Bl/6J mice received a normal diet, high-fat diet (HFD), or HFD with K21 administration (10^9^ CFU in 0.2 mL PBS/day) for eight weeks. Supplementation of K21, but not placebo, appeared to alleviate body weight gain and epididymal fat mass accumulation, reduce plasma leptin levels, decrease cholesterol and triglyceride levels, and mitigate liver damage in DIO mice. Moreover, the hepatic expression of peroxisome proliferator-activated receptor-*γ* (PPAR-*γ*) related to adipogenesis was significantly downregulated in DIO mice by K21 intervention. We also found that K21 supplementation strengthens intestinal permeability and modulates the amount of *Lactobacillus* spp., *Bifidobacterium* spp., and *Clostridium perfringens* in the cecal contents of DIO mice. In conclusion, our results suggest that dietary intake of K21 protects against the onset of HFD-induced obesity through multiple mechanisms of action.

## 1. Introduction 

Metabolic syndrome is a common metabolic disorder that results from the increasing prevalence of obesity [1]. It combines disturbances in glucose and insulin metabolism, excess weight, mild dyslipidemia, a proinflammatory state, hypertension, subsequent development of type 2 diabetes (T2D), nonalcoholic fatty liver disease (NAFLD), and cardiovascular disease [1–3]. Accumulating evidence indicates that the gut microbiota is associated with the development of obesity and related metabolic disorders [4–6]. Moreover, colonization of germ-free mice with gut microbes from obese mice results in a greater weight gain and body fat accumulation than colonization with that from lean mice [7, 8], indicating the importance of gut microbiota on host metabolism.

The modulation of gut microbiota affects host metabolism and has an impact on energy homeostasis and ectopic fat deposition [9]. The advent of probiotic treatments appears to be a promising approach to reverse the dysbiosis-linked host metabolic alterations observed in obesity and related disorders [10]. Probiotics are defined as living microorganisms that, when administered in adequate amounts, confer health benefits to the host [11]. Most microorganisms identified to date as probiotics are Gram-positive bacteria and belong to the genera* Lactobacillus* or* Bifidobacterium,* which have been used for centuries because of their benefits to human health [12]. Although the molecular mechanism of probiotic action is not fully elucidated, many effects may prove beneficial in obesity and in related disorders; these effects include the modulation of the intestinal microbiota, modification of the local microenvironment inside the gut, enhancement of the epithelial barrier function, and the regulation of the host immune response [13, 14].

In this study, we aimed to identify probiotic strains with potential antiobesity activities by* in vitro* assays, and a specific* Lactobacillus plantarum* strain K21 was thus identified. Moreover, K21 was found to inhibit lipid accumulation in 3T3-L1 preadipocytes. To further investigate if K21 possesses antiobesity effects* in vivo*, a diet-induced obese (DIO) mouse model was used. Male C57Bl/6J mice were fed with a Western diet, to induce obesity and obesity-related disorders; the mice were simultaneously supplemented with K21, to examine its effects on the progression of obesity, hyperlipidemia, liver damage, and hyperglycemia.

## 2. Materials and Methods

### 2.1. Bacterial Strain, Media, and Growth Conditions

*L. plantarum* K21 was isolated from locally fermented vegetables and preserved in our lactic acid bacterial bank. K21 was statically grown in Man Rogosa Sharpe (MRS; BD Difco, Franklin Lakes, NJ) broth at 37°C for 18–20 h. For* in vivo* assays, the K21 culture was harvested using centrifugation (1500 ×g, 10 min), washed twice with sterile PBS, and resuspended to a final concentration of approximately 10^10^ CFU/mL.

### 2.2. Bile Salt Hydrolyzing Ability

The bacterial ability to hydrolyze bile salt was assessed by performing a plate assay [15]. Bile salt plates were prepared using 0.5% (w/v) taurodeoxycholic acid sodium salt (TDCA; Sigma, St Louis, MO) and 0.037% (w/v) CaCl_2_ in MRS plate [15]. Briefly, bacterial cultures grown overnight were, respectively, inoculated in MRS and bile salt plates. The inoculated plates were incubated anaerobically at 37°C for 72 h, and the colonial morphology was observed. A bacterial strain with ability to hydrolyze TDCA resulted in white precipitates on the bile salt plate but not on the MRS plate, while strains without the ability produced similar colony types on MRS and bile salt plates. The result was reproduced in triplicate in three independent experiments.

### 2.3. Cholesterol-Lowering Ability

The ability of bacterial cultures to assimilate cholesterol was determined using a modified method described by Danielson et al. [16]. Each freshly prepared strain culture was inoculated (1%) in 1 mL of MRS broth supplemented with 0.2% sodium thioglycollate (Sigma), 0.3% oxgall (BD Difco, Franklin Lakes, NJ), and a water-soluble form of cholesterol (cholesterol-methyl-*β*-cyclodextrin, Sigma). The final concentration of cholesterol in the broth was 60 mg/dL. The tubes were anaerobically incubated at 37°C for 24 h. After incubation, cells were centrifuged for 10 min at 12,000 ×g, at 4°C. The amounts of cholesterol in the spent broth and uninoculated sterile broth, as a negative control, were determined using an enzymatic method, according to the manufacturer's instructions (Randox, Crumlin, UK).

### 2.4. Preadipocytes Differentiation Assay

3T3-L1 cell culture, differentiation, and Oil Red staining were performed as previously described [17]. Briefly, 3T3-L1 cells were cultured in 12-well plates (6 × 10^4^/well) in high-glucose DMEM containing 10% FBS, 100 U/mL penicillin, and 0.1 mg/mL streptomycin. After confluence (day 0), the cells were stimulated in a differentiation medium (DM) containing 10% FBS, 0.5 mM IBMX, 0.5 *μ*M dexamethasone, and 10 *μ*g/mL insulin, in the presence or absence of heat-killed K21 (10^7^ CFU/mL). After 3 days of stimulation (day 3), the media were replaced by 10% FBS/DMEM containing 10 *μ*g/mL insulin. On day 9, the intracellular lipids of the 3T3-L1 cells were stained with Oil Red-O and observed by a previously described method [17].

### 2.5. Animals, Diets, and Experimental Design

In all, 24 male C57Bl/6J mice (8 weeks old) were purchased from the National Laboratory Animal Center (NLAC; Taipei, Taiwan) and housed in a controlled environment (22°C, 50−60% humidity, and 12 h light/dark cycle) with free access to food and water. The mice were randomly selected and assigned to three groups (eight mice per group) according to the type of diet and test-material. Group 1 (normal diet, ND) was fed with a standard chow diet (Autoclavable Rodent Diet 5010, low fat diet, 12% energy from fat, LabDiet) and placebo (0.2 mL PBS via an orogastric tube daily); group 2 (high-fat-diet, HFD) with HFD (Western diet 5TJN, 40% of energy from fat with 0.15% cholesterol, TestDiet) and placebo; and group 3 (HFD + K21) with HFD and* L. plantarum* K21 (approximately 10^9^ CFU/0.2 mL via an orogastric tube daily) for eight weeks. All the animal experimental procedures followed the guidelines of Care and Use of Laboratory Animals, and the study was approved by the Animal Care and Ethics Committee of the National Yang-Ming University. Mice were sacrificed, and the livers were rapidly excised and fixed in 10% neutral-buffered formalin for paraffin-embedded sections and hematoxylin and eosin staining.

### 2.6. Blood Chemistry

Blood plasma was analyzed using the FUJI DRI-CHEM SYSTEM 3500s (Fuji Photo Film CO. Ltd., Tokyo, Japan) for measurement of glucose, cholesterol, NEFA (nonesterified fatty acids), triglycerides, aspartate aminotransferase (AST), and alanine aminotransferase (ALT). Plasma leptin and adiponectin were determined using ELISA kits (Boster, Wuhan, China); plasma insulin was determined using an ELISA kit (Mercodia, Uppsala, Sweden), according to the manufacturer's instructions.

### 2.7. Quantitative Real-Time PCR (qRT-PCR)

Total RNA from each mice liver was prepared using the RNeasy mini kit (Qiagen, Hilden, Germany), and the cDNA was then synthesized using an oligo (dT) 15 primer and SuperScript II reverse transcriptase reagents (Invitrogen, Carlsbad, CA). Subsequently, qRT-PCR was performed in a LightCycler instrument (Roche, Basel, Switzerland) using the DyNAmo Capillary SYBR Green qPCR kit (Finnzymes, Espoo, Finland), as per the manufacturer's recommendations. The forward primer 5′-TGC TGT TAT GG GTG AAA CTC TG-3′ and the reverse primer 5′-GAA ATC AAC TGT GGT AAA GGG C-3′ were used to detect PPAR-*γ* (GenBank number: NM_011146), whereas the forward primer 5′-GTA TGA CTC CAC TCA CGG CAA A-3′ and the reverse primer 5′-GGT CTC GCT CCT GGA AGA TG-3′ were used to detect GAPDH (GenBank number: NM_008084). The thermal cycling conditions were 6 min at 95°C, followed by 50 cycles of denaturation at 95°C for 10 s, annealing at 57°C for 10 s, and extension at 72°C for 8 s. The expression levels of the mRNAs of each sample were normalized, with GAPDH serving as an internal control. The results were expressed as relative expression ratios with respect to the control group.

### 2.8. Intestinal Permeability* In Vivo*

This measure is based on the intestinal permeability towards the 4 kDa fluorescent dextran–FITC (DX-4000–FITC) (Sigma) as described [18]. Briefly, mice that had fasted for 12 h were given DX-4000–FITC by gavage (500 mg/kg body weight, 50 mg/mL). After 1 h, 120 *μ*L of blood was collected from the tip of the tail vein. The blood was centrifuged at 12 000 ×g for 3 min at 4°C. The plasma was diluted in an equal volume of PBS and analyzed for the DX-4000–FITC concentration with a fluorescence spectrophotometer (Tecan, Männedorf, Switzerland) using an excitation wavelength of 485 nm and emission wavelength of 535 nm. Standard curves were obtained by diluting the FITC–dextran in nontreated plasma diluted with PBS (1 : 3 v/v).

### 2.9. Analysis of Cecal Microflora

After the mice were sacrificed, the cecum of each animal was removed and 0.1 g of the cecal content was weighed, transferred into a tube with 0.9 mL of anaerobic diluent, and homogenized by vortexing. The homogenates (diluted with appropriate dilution factors) were plated in selective culture medium in triplicate. MRS agar was used for* Lactobacillus *spp., BIM-25 agar was used for* Bifidobacterium *spp., and egg yolk-free TSC (tryptose-sulfite-cycloserine) agar was used for* Clostridium perfringens *[19]. Subsequently, the plates were anaerobically incubated at 37°C for 48 h for CFU counting.

### 2.10. Statistical Analysis

The statistical significance was determined using Prism5 (GraphPad, San Diego, CA), using one-way ANOVA followed by the Bonferroni post hoc correction. A *P* value of <0.05 was considered significant in all cases. All the assays were reproduced with at least three independent experiments. Each sample was assayed in triplicate, and the mean activity and the standard deviation (SD) were determined.

## 3. Results

### 3.1. *In Vitro* Screening for Lactic Acid Bacteria with Potential Antiobesity Activity

**Figure 1:**
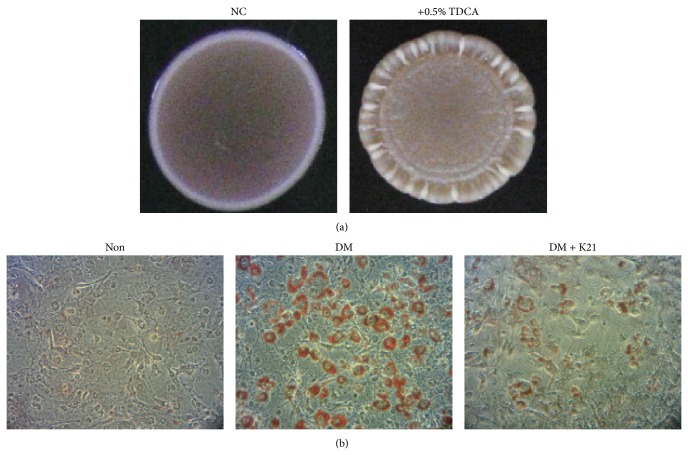
Qualitative assessment of* L. plantarum* K21 for hydrolyzing bile salt and inhibiting lipid accumulation in 3T3-L1 preadipocytes. (a) Demonstration of the ability of K21 to hydrolyze bile salt. The K21 strain was inoculated on an MRS agar plate (NC, negative control) or an MRS agar plate containing 0.5% TDCA, as indicated. The plates were incubated at 37°C under anaerobic conditions for three days, and the colonial morphology was observed. In the assays shown, precipitation in the agar is indicative of bacterial ability for hydrolyzing TDCA. (b) The inhibitory effects of K21 on lipid accumulation in 3T3-L1 preadipocytes. 3T3-L1 cells were differentiated with differentiation medium (DM) in the absence or presence of heat-killed K21 (~10^7^ CFU/mL) as described in Materials and Methods. Intracellular lipids were stained with Oil Red-O. Non, undifferentiated cells. The results were confirmed with three independent experiments.

To identify potential probiotic strains with antiobesity activity, we performed qualitative assays to screen for isolates with abilities to hydrolyze bile and cholesterol, both of which have been correlated with antiobesity effects [20]. A total number of 88 lactic acid bacterial strains collected in our laboratory were screened. Among these strains, 50 and 27 strains were, respectively, found to harbor abilities required for hydrolyzing the conjugated bile acid, TDCA, on the MRS agar plate and reducing cholesterol (>50% reduction) in the MRS broth. A specific* L. plantarum* strain, K21, with abilities to hydrolyze TDCA (Figure 1(a)) and cause ~65% reduction of cholesterol in the MRS broth was thus selected for further analyses. We also investigated the effects of K21 on 3T3-L1 preadipocyte differentiation. The result showed that 3T3-L1 preadipocytes stimulated with differentiation medium (DM) resulted in an increased intracellular lipid accumulation, as assessed by Oil Red-O staining, which could be apparently inhibited by the treatment of K21 (Figure 1(b)).

### 3.2. *L. plantarum *K21 Inhibits Body Weight Gain and Fat Weight Accumulation in DIO Mice

**Figure 2:**
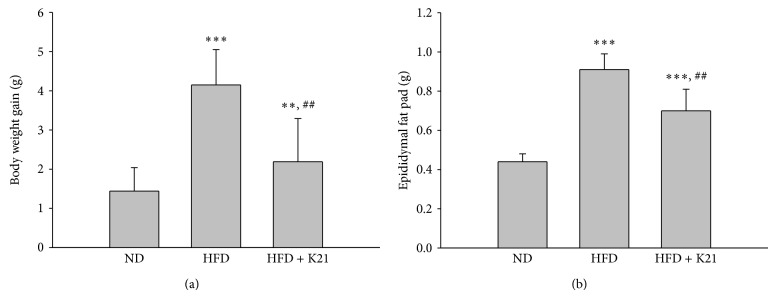
*L. plantarum* K21 supplementation decreases HFD-induced body weight gain and fat mass accumulation. (a) Change in body weight gain; (b) weight of epididymal fat pad. Values are means with SD. ^**^ *P* < 0.01, ^***^ *P* < 0.001* versus* the ND group; ^##^ *P* < 0.01* versus* the HFD group.

To investigate if K21 possesses antiobesity activities* in vivo*, mice were fed with normal diet (ND group), high-fat diet (HFD group), or HFD supplemented with K21 (HFD + K21) (10^9^ CFU in 0.2 mL PBS/mouse/day) for eight weeks, the ND and HFD groups were fed with placebo (0.2 mL PBS/mouse/day), and their body weight was monitored. After 8-week feeding, the HFD group showed significantly increased body weight (*P* < 0.01) compared with that of the ND group (Table 1). K21 supplementation ameliorated the increased body weight of HFD-fed mice, although the difference was not statistically significant (Table 1). As shown in Figure 2(a), the body weight gain of the HFD group was also increased (*P* < 0.001) compared to the ND group, and the K21 supplementation attenuated this increase (*P* < 0.01); however, the body weight gain of the HFD + K21 group is still higher (*P* < 0.01) than that of the ND group. These results revealed that the supplementation of K21 moderately attenuated the increased body weight of HFD-fed mice. On the other hand, the epididymal fat mass was significantly higher in the HFD and HFD + K21 groups than in the ND group (*P* < 0.001) (Figure 2(b)). As compared to HFD-fed mice, the K21-treated mice showed a significant reduction in the epididymal fat mass (*P* < 0.05). In addition, the food efficiency ratio (FER), total grams of body weight gained on a test food divided by the total grams of food consumed during an animal feeding study, of the HFD-fed mice was significantly increased compared with that of the ND-fed mice (*P* < 0.001), suggesting greater efficiency of HFD on weight gain (Table 1). On the contrary, the supplementation of K21 reduced the FER of HFD-fed mice (*P* < 0.01), reflecting lower weight gain per grams of food consumed. Although the amount of food intake was significantly reduced in the HFD and HFD + K21 groups compared to the ND group, the supplementation of K21 did not influence the amount of food intake in HFD-fed mice (Table 1).

### 3.3. *L. plantarum* K21 Attenuates Dyslipidemia in DIO Mice

As shown in Table 1, the plasma cholesterol and triglyceride levels in the HFD group showed a significant increase (*P* < 0.001), suggesting the onset of dyslipidemia by HFD feeding. K21 supplementation was found to significantly mitigate the cholesterol and triglyceride levels (*P* < 0.05 and *P* < 0.001, resp.). The serum NEFA levels were not significantly different among the three groups. In addition, HFD significantly increased the leptin level (*P* < 0.001), which was ameliorated by the K21 supplementation (*P* < 0.001), although the leptin level of the HFD + K21 group was still higher than that of the ND group (*P* < 0.001). Compared with the ND group, the HFD group showed a decreased level of adiponectin (*P* < 0.01), while no statistically significant difference was found between the HFD + K21 group and the other two groups. On the other hand, the fasting plasma glucose and insulin levels were elevated more in the HFD group than in the ND group (*P* < 0.001); however, the K21 supplementation did not suppress these effects (Table 1).

### 3.4. *L. plantarum* K21 Attenuates Liver Damage and Regulates Hepatic PPAR-*γ* Expression in DIO Mice

**Figure 3:**
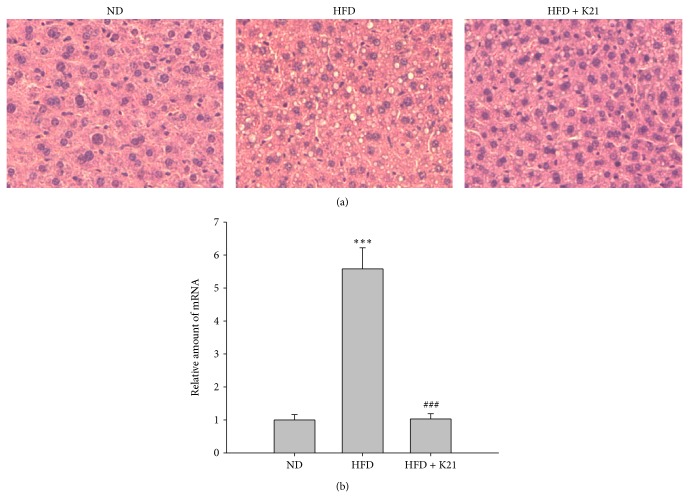
*L. plantarum *K21 attenuates liver damages and hepatic mRNA expression of PPAR-*γ* in DIO mice. (a) Effect of K21 on hepatic histology of mice fed with HFD for eight weeks. The microphotographs of liver tissue sections were analyzed with H&E staining at 200x. (b) qRT-PCR analysis of the hepatic mRNA expression of PPAR-*γ*. The mRNA expression of GAPDH was used as an internal control for data normalization. Values are means with SD. ^***^ *P* < 0.001 versus the ND group; ^###^ *P* < 0.001 versus the HFD group.

As shown in Figure 3(a), the liver histology of the HFD group (unlike the ND group), showed clear evidence of micro- and macrovesicular steatosis resulting from the accumulation of fat droplets, which could be ameliorated by the K21 supplementation. To further characterize steatosis in mice, the plasma levels of aspartate AST and ALT (markers of hepatic dysfunction) were measured. As shown in Table 1, the AST and ALT levels were significantly higher (*P* < 0.001 and *P* < 0.01, resp.) in the HFD group than in the ND group, and K21 supplementation could mitigate the elevated levels of AST and ALT (*P* < 0.001 and *P* < 0.05, resp.) induced by HFD feeding (Table 1). In addition, hepatic cholesterol and triglyceride were obviously increased (*P* < 0.001) in the HFD group, which could be ameliorated by the supplementation of K21 (Table 1). We also measured the hepatic mRNA levels of PPAR-*γ*, which is an important regulator of lipid homeostasis. The expression of PPAR-*γ* is generally increased in steatotic livers [21]. As shown in Figure 3(b), the mRNA levels of PPAR-*γ* were found to significantly increase in the HFD group (*P* < 0.001) than in the ND group, and the administration of K21 significantly normalized this effect (*P* < 0.001).

### 3.5. *L. plantarum* K21 Attenuates Increased Gut Permeability in DIO Mice

**Figure 4:**
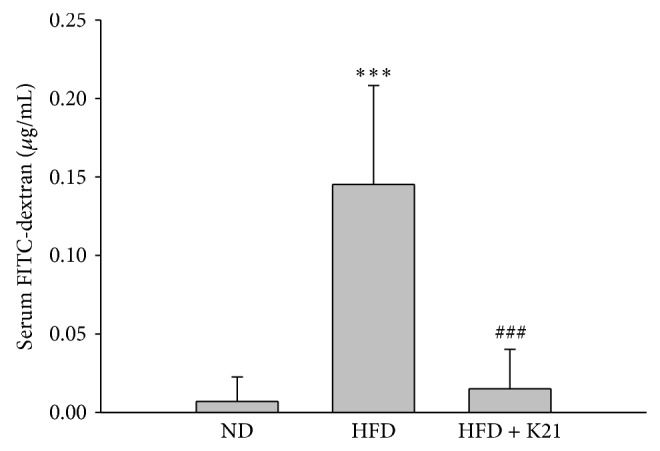
*L. plantarum *K21 attenuates the increased intestinal permeability in DIO mice. Serum levels of 4-kDa FITC-dextran were determined one hour after oral gavage in mice at eight weeks as described in Materials and Methods. Values are means with SD. ^***^ *P* < 0.001 versus the ND group; ^###^ *P* < 0.001 versus the HFD group.

Diet-induced obesity has been correlated to increased intestinal permeability [22, 23], and specific probiotics with beneficial effects on the gut barrier functions have been reported [24]. In order to study the effect of K21 supplementation on the gut barrier functions in DIO mice, the intestinal permeability was measured using FITC-dextran. The serum levels of FITC-dextran were higher in the HFD group compared to the ND group (*P* < 0.001) (Figure 4), thereby indicating an increased intestinal permeability in DIO mice. K21 supplementation clearly attenuated this increase (*P* < 0.001).

### 3.6. *L. plantarum* K21 Modulates Members of the Cecal Microbiota

**Figure 5:**
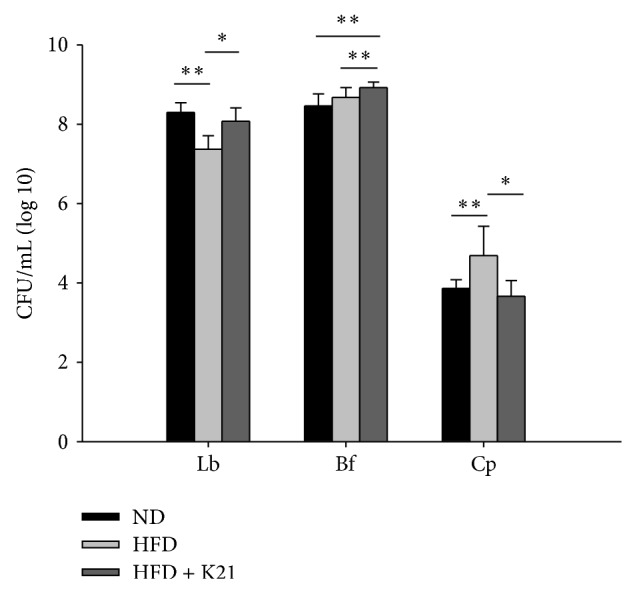
HFD and probiotic modulate members of the cecal microbiota in mice. The amount of* Lactobacillus* spp. (Lb),* Bifidobacterium *spp. (Bf), and* C. perfringens* (Cp) in the ceca of mice at eight weeks were determined as described in Materials and Methods. Values are means with SD. ^*^ *P* < 0.05 and ^**^ *P* < 0.01 between the indicated groups.

Specific probiotics are known to modulate the gut microbiota composition, by increasing beneficial and decreasing harmful bacteria, to promote human health. To determine whether HFD-feeding and K21 supplementation modulate members of the gut microbiota in mice,* Lactobacillus* spp. and* Bifidobacterium* spp., which are generally regarded as beneficial bacteria, and a potential gut pathogen* C. perfringens* were measured from cecal contents by plate counting method. As shown in Figure 5,* Lactobacillus* spp. was decreased in the HFD group than in the ND group (*P* < 0.01), and K21 supplementation attenuated this effect (*P* < 0.05). Unlike the HFD- and ND-fed mice, the K21-supplemented mice showed a significant increase in the cecal amount of* Bifidobacterium* spp. (*P* < 0.01). The levels of* C. perfringens* were increased with HFD-feeding (*P* < 0.01), but this effect could be attenuated by K21 supplementation (*P* < 0.05).

## 4. Discussion

Clinical and experimental studies suggest that probiotics differ greatly in their efficacy and mechanisms of action [10]. In an effort to treat obesity and obesity-related disorders, numerous probiotic strains have been evaluated for their ability to modulate gut microbiota [25], lower serum cholesterol and triglycerides [26], decrease fat storage [27], reduce adipocyte size [28, 29], improve insulin resistance, decrease glucose intolerance [30], and ameliorate liver dysfunction in chronic liver disease [24, 31]. It is well-known that most effects of probiotics are strain-specific and cannot be extended to other probiotics of the same genus or species. However, a meta-analysis study has indicated that the effect of* Lactobacillus* spp. on weight is species dependent [32]. The administration of* Lactobacillus acidophilus*,* Lactobacillus fermentum*, and* Lactobacillus ingluviei *is associated with weight gain, whereas the administration of* L. plantarum *and* Lactobacillus gasseri *is associated with weight loss in obese humans and animals [32]. In our study, supplementation with K21, belonging to* L. plantarum*, inhibited weight gain in DIO mice, which also supported the finding of the meta-analysis mentioned above. Moreover, compared to other studies on similar probiotic strains, K21 appeared to confer beneficial effects on DIO mice through multiple mechanisms of action.

BSH activity has been mostly observed in Gram-positive commensals but generally not in the Gram-negative commensals of the gastrointestinal tract [20]. The presence of BSH in probiotics helps reduce the blood cholesterol level in the host, which also renders them more tolerant to bile salts [20]. As shown in Figure 1(a), K21 appeared to harbor an* in vitro* BSH activity and inhibited the blood cholesterol level in DIO mice (Table 1). We also found that K21 possessed a higher tolerance against 0.5% oxgall than did the two well-studied probiotic strains, namely,* L. casei* Shirota and* L. rhamnosus* GG (data not shown). BSHs are very specific to certain bile types, and their duration of contact with bile ensures bacterial survival in varying bile environments [20]. In the genome of* L. plantarum* WCFS1, four* bsh* genes were identified, and these may confer additional advantages to the bacterial persistence in the gut [33]. Whether K21 harbors multiple genes encoding BSHs remains to be studied.

Our results indicate that K21 supplementation ameliorates metabolic alterations in DIO mice, including such parameters as obesity, liver damages, and glucose intolerance. We also found that an increased expression of hepatic PPAR-*γ* mRNA was reversed in response to K21 intervention in DIO mice (Figure 3(b)). PPAR-*γ* is an important regulator of lipid homeostasis [34], and it expresses most abundantly in the adipose tissue, where it plays a role in increasing insulin sensitivity [35]. Increased PPAR-*γ* mRNA levels are usually observed in steatotic livers [21]. Thus, K21 may induce metabolic alterations in the livers of DIO mice by regulating the PPAR-*γ* expression. Previous reports have also described an elevated expression of PPAR-*γ* in the livers of DIO mice [25, 36]. Probiotic treatment attenuates liver steatosis in DIO mice but causes a superinduction of hepatic PPAR-*γ* activities [25, 36]. Elevated PPAR*γ* levels may decrease the production of proinflammatory cytokines in order to reduce hepatic inflammation [37]. These findings suggested that probiotics exert differential mechanisms of action, although they produce similar effects.

Probiotics may provide a way to alter the gut microbiota naturally, which may partly explain the modulation of host metabolism in response to probiotic interventions [9]. As shown in Figure 5, the supplementation of K21 increased the amount of cecal* Lactobacillus* spp. and* Bifidobacterium* spp. but decreased the amount of* C. perfringens*, suggesting that K21 modulates the gut microbiota in DIO mice, which may result into antiobesity effects. Consistent with our finding, supplementation of* Lactobacillus curvatus* HY7601 and* L. plantarum *KY1032 increased the endogenous* Bifidobacterium pseudolongum* in DIO mice, which appears to be correlated with the suppression of body weight gain or body fat reduction [25]. However, it has also been described that* L. acidophilus* NCDC 13 supplementation significantly increases the amount of* Bifidobacterium* spp. in both fecal and cecal contents, with no detectable effect on obesity parameters in DIO mice [38]. Thus, the correlation between the modulation of gut microbiota by probiotic interventions and its influence on host metabolism warrants further investigations.

In conclusion, the probiotic* L. plantarum* K21, which harbors bile salt hydrolyzing and cholesterol-lowing abilities, inhibits the differentiation of preadipocytes. In DIO mice, supplementing K21 inhibits body weight gain and fat mass accumulation, decreases the level of leptin, reduces liver damage, and inhibits the hepatic expression of PPAR-*γ*. Furthermore, K21 also strengthens intestinal barrier functions and modulates the gut microbiota. These results provide an opportunity to develop foods and identify other LAB strains with potential antiobesity activity through* in vitro *screening assays. Furthermore, our findings show that* L. plantarum* strain K21 helps alleviate obesity and obesity-related metabolic syndrome in a mouse model, and clinical trials to confirm these effects in humans should hence be conducted in the near future.

## Figures and Tables

**Table 1:** Effects of experimental diets and K21 administration on mice.

Parameters assessed	ND	HFD	HFD + K21
Initial body weight (g)	22.56 ± 1.46	22.51 ± 1.30	22.61 ± 1.79
Final body weight (g)	24.00 ± 1.2	26.66 ± 1.74^**^	24.80 ± 1.8
Food intake (g/mouse/day)	2.72 ± 0.04	2.18 ± 0.12^***^	1.97 ± 0.25^***^
Food efficiency ratio (%)	1.04 ± 0.26	4.13 ± 0.55^***^	2.81 ± 0.63^∗∗∗,##^
Cholesterol (mg/dL)	78.7 ± 9.7	170.2 ± 16.5^***^	148.7 ± 15.4^∗∗∗,#^
Triglyceride (mg/dL)	73.8 ± 8.3	104.5 ± 5.3^***^	70.6 ± 8.8^###^
NEFA (mmol/L)	1.51 ± 0.25	1.39 ± 0.27	1.20 ± 0.42
Leptin (ng/mL)	638.8 ± 179.8	3670.8 ± 1272.7^***^	1616.4 ± 393.7^∗∗∗,###^
Adiponectin (ng/mL)	26.5 ± 2.4	22.6 ± 2.4^**^	24.3 ± 2.7
Glucose (mg/mL)	104.8 ± 19.9	189.2 ± 16.9^***^	175.0 ± 33.1^***^
Insulin (mg/mL)	14.07 ± 9.37	149.44 ± 28.35^***^	98.87 ± 47.99^*^
Liver weight (g)	1.00 ± 0.05	0.95 ± 0.06	0.99 ± 0.03
AST (U/L)	66.6 ± 18.9	285.5 ± 32.1^***^	74.4 ± 18.6^###^
ALT (U/L)	32.5 ± 5.0	61.8 ± 22.7^**^	39.2 ± 14.6^#^
Hepatic cholesterol (mg/dL)	44.2 ± 4.3	108.2 ± 26.2^***^	81.3 ± 20.5^∗∗∗,#^
Hepatic triglyceride (mg/dL)	161.2 ± 30.6	244.1 ± 47.8^***^	134.0 ± 26.8^###^

C57Bl/6J mice were fed a normal diet (ND), a high-fat diet (HFD), or a HFD + *L*. *plantarum* K21 (10^9^ CFU per day) for eight weeks. Data represent the means ± SD of eight mice per group. ^*^ *P* < 0.05, ^**^ *P* < 0.01, and ^***^ *P* < 0.001 versus the ND group; ^#^ *P* < 0.05, ^##^ *P* < 0.01, and ^###^ *P* < 0.001 versus the HFD group.
